# Plasma heme-induced renal toxicity is related to a capillary rarefaction

**DOI:** 10.1038/srep40156

**Published:** 2017-01-10

**Authors:** Nahid Tabibzadeh, Céline Estournet, Sandrine Placier, Joëlle Perez, Héloïse Bilbault, Alexis Girshovich, Sophie Vandermeersch, Chantal Jouanneau, Emmanuel Letavernier, Nadjib Hammoudi, François Lionnet, Jean-Philippe Haymann

**Affiliations:** 1 Sorbonne Universités UPMC Univ Paris 06, Paris, France; 2 Service d’Explorations Fonctionnelles Hôpital Tenon, Assistance Publique-Hôpitaux de Paris, Paris, France; 3 Institut National de la Sante et de la Recherche Médicale, UMR-S 1155, Paris, France; 4 Ecole Vétérinaire de Maisons Alfort, France; 5 Institut de Cardiologie, Pitié-Salpêtrière Hospital (AP-HP), Institute of Cardiometabolism and Nutrition (ICAN), ACTION Study Group, INSERM UMRS 1166, Paris, France; 6 Centre de la drépanocytose, service de médecine interne, Paris, France

## Abstract

Severe hypertension can lead to malignant hypertension (MH) with renal thrombotic microangiopathy and hemolysis. The role of plasma heme release in this setting is unknown. We aimed at evaluating the effect of a mild plasma heme increase by hemin administration in angiotensin II (AngII)-mediated hypertensive rats. Prevalence of MH and blood pressure values were similar in AngII and AngII + hemin groups. MH rats displayed a decreased renal blood flow (RBF), increased renal vascular resistances (RVR), and increased aorta and interlobar arteries remodeling with a severe renal microcirculation assessed by peritubular capillaries (PTC) rarefaction. Hemin-treated rats with or without AngII displayed also a decreased RBF and increased RVR explained only by PCT rarefaction. In AngII rats, RBF was similar to controls (with increased RVR). PTC density appeared strongly correlated to tubular damage score (rho = −0.65, p < 0.0001) and also renal Heme Oygenase-1 (HO-1) mRNA (rho = −0.67, p < 0.0001). HO-1 was expressed in PTC and renal tubules in MH rats, but only in PTC in other groups. In conclusion, though increased plasma heme does not play a role in triggering or aggravating MH, heme release appears as a relevant toxic mediator leading to renal impairment, primarily through PTC endothelial dysfunction rather than direct tubular toxicity.

Heme is an essential component of several proteins in the organism including hemoglobin, mitochondrial cytochromes and NO synthase. Whereas a low heme synthesis is responsible for porphyria^1^, heme intracellular accumulation is likely to be also deleterious through induction of intracellular oxidative stress as encountered in some ischemic and toxic conditions^2^,^3^. Of notice, circulating heme (free or bound to hemopexin) may also lead to endothelial and renal tubular toxicity^4^,^5^,^6^,^7^,^8^. During hemolysis, substantial amounts of heme are indeed released by red blood cells, and form a heme-hemopexin complex which binds to its receptor CD91 on endothelial or renal tubular cell surface, leading to subsequent endocytosis, intracellular accumulation of heme and iron store leading ultimately to ROS generation^9^. Indeed, heme toxicity is considered to be responsible for sickle cell anemia nephropathy (SCAN) through renal microcirculation impairment^10^,^11^,^12^ and cases of acute renal failure following administration of hematin for acute intermittent porphyrias were reported^13^,^14^. Heme metabolization thus appears as a critical step to ensure cytoprotection. Heme-oxygenase 1 (HO-1) is an inducible enzyme which catabolizes heme, through an enzymatic reaction which produces biliverdin (subsequently converted to bilirubin), carbon monoxide and iron^15^. The ability to induce HO-1 within renal tissue appears a critical event for nephroprotection^16^,^17^ especially in the setting of vascular injuries^18^ or heme overload as illustrated by mice lacking HO-1 who develop more severe lesions after ischemia/reperfusion^19^. Accordingly, multi-organ failure including renal failure, and severe endothelial lesions were reported in a child with an inactivating mutation of HO-1^20^. We thus speculated that in severe hypertensive subjects related to activation of the renin-angiotensin-aldosterone system, the onset of a mild hemolysis would be a critical event leading to an imbalance between heme overload and HO-1 induction and thus would trigger malignant hypertension.

Our results ruled out our working hypothesis but showed surprisingly that a mild rise of plasma heme for two weeks impaired renal blood flow through renal capillary rarefaction with no significant influence of angiotensin II mediated hypertension within this time frame.

## Results

**Figure 1:**
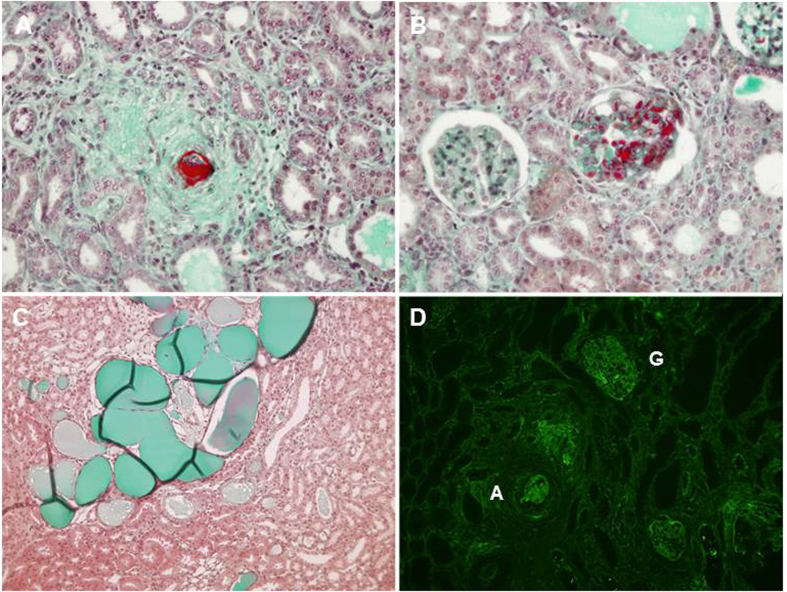
Renal histology of MH rats at day 28. Fibrinoid necrosis, hyperplastic arteriolosclerosis and onion-skin thickening of the arteriolar wall (**A**). Arteriolar (**A**) and glomerular capillary (**B**) thrombosis. Acute tubular necrosis (**C**). Fibrin deposits (immunofluorescent staining) (**D**). A: arteriole, G: glomerulus.

**Figure 2:**
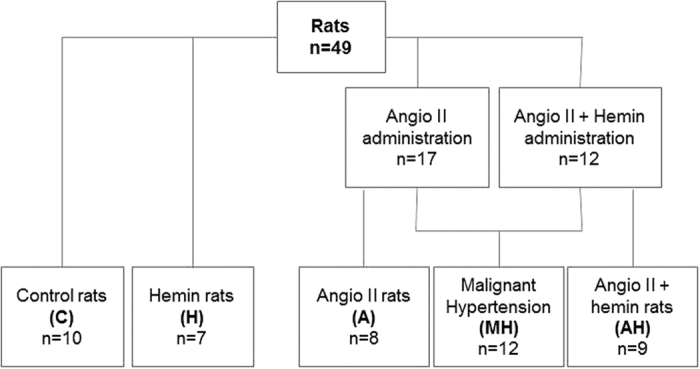
Experimental flow chart. C: control group, H: hemin group, A: angiotensin II group, AH: angiotensin II + hemin group, MH: malignant hypertension group. Angiotensin II infusion by a minipump (or vehicle) (400 ng/kg/min) was performed for 28 days. Hemin (or vehicle) was given (ip) from day 14 to day 28 (50 mg/kg), thrice a week.

**Figure 3:**
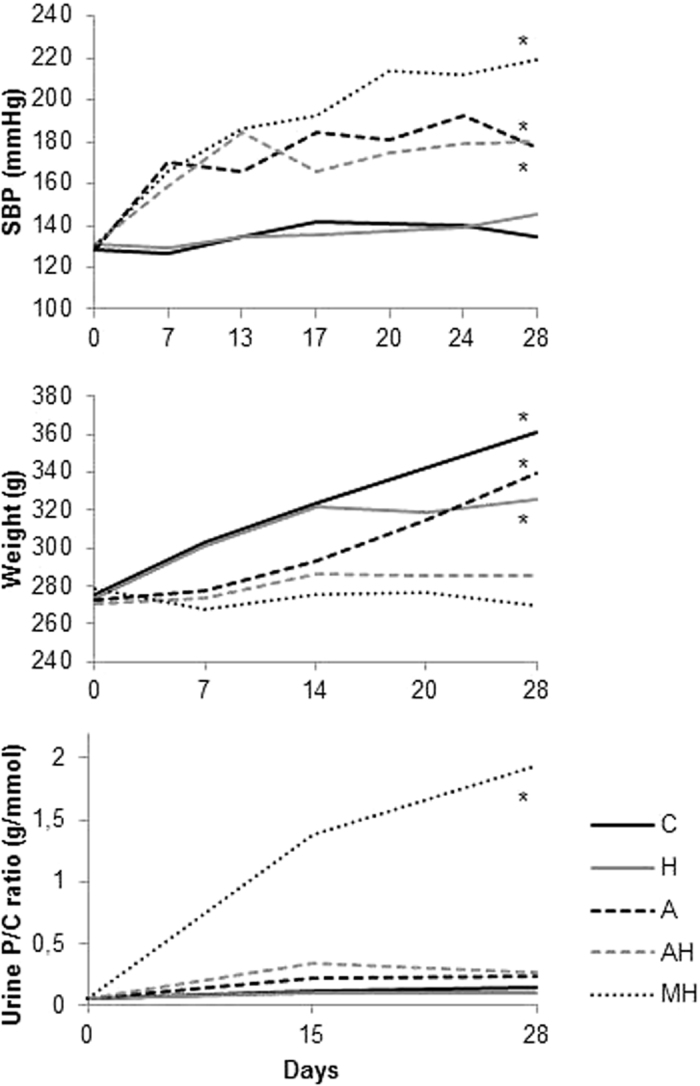
Blood pressure, weight and proteinuria during the study. SBP: systolic blood pressure, P/C: proteinuria/creatinuria ratio, C: controls, H: hemin group, A: angiotensin II group, AH: angiotensin II + hemin group, MH: malignant hypertension group. Asterisks indicate a p value < 0.05 compared to control rats.

Among the 29 rats receiving angiotensin II, 45% developed malignant hypertension (MH) assessed by histological hallmark features, illustrated Fig. 1, such as proliferating endarteritis with arteriolar thrombosis, fibrinoid necrosis in vessel walls and/or glomerular capillary vessels (assessed by fibrin deposits). Indeed, necrosis was present in 53.7% +/−5.7 of vessels and 63.2% +/− 3.8 of glomeruli in MH group samples with no necrosis detected in the other groups. In rats receiving angiotensin II alone, 53% (n = 9) developed MH, whereas MH occurred in 25% of rats receiving angiotensin II and Heme (NS). Thus, angiotensin II treated animals with no MH were classified as group Ang II (A) or Ang II + heme (AH) accordingly (flow chart see Fig. 2). Under Ang II treatment, systolic blood pressure increased as soon as day 7 and further increased up to day 28, with no significant difference in hemin treated group (Fig. 3). Moreover whereas blood pressure values were similar in control and group H, animals from group H experienced an average loss of 14% of body weight compared to controls after initiation of hemin (between day 14 and 28) (p < 0.01). Similarly, AH and MH groups experienced an average 15% loss compared to A group (p < 0.01 and 0.01 respectively). Proteinuria assessed by urinary protein/creatinine ratio increased at day 15 and 28 in MH group only, with no significant difference between the two time points. At day 28, plasma heme was increased in rats receiving heme (H and AH rats, p = 0.002), with a mild increase detected in group A (Table 1). Moreover, MH rats had a higher plasma heme and bilirubin concentration than control group (p = 0.03 and 0.004) and also a decreased haematocrit (p = 0.03).

**Figure 4:**
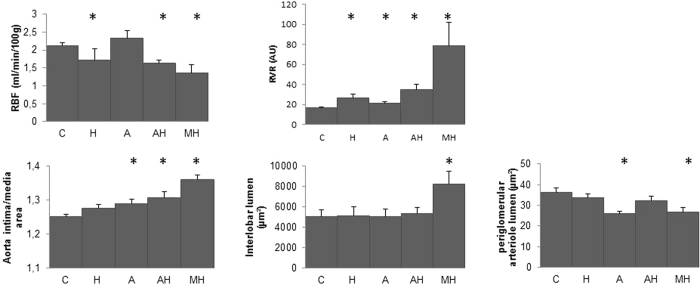
Renal hemodynamic and vessels morphometry evaluation at day 28. Values expressed in Mean [SEM]. RBF: renal blood flow, RVR: renal vascular resistance (AU: arbitrary units), C: controls, H: hemin rats, A: angiotensin II rats, AH: angiotensin II and hemin rats, MH: malignant hypertension. The asterisk indicates a p value < 0.05 compared to control rats.

**Figure 5:**
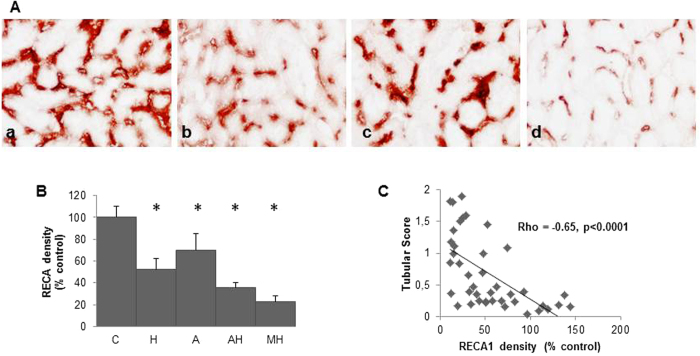
Peritubular capillary density evaluation at day 28. (**A**) RECA1 staining in kidneys of control rats (a), hemin rats b), angiotensin II rats (c) and angiotensin II + hemin rats (d). Magnification X 600. (**B**) Quantification of RECA1 staining according to the different groups (data are expressed as % of controls). Values expressed in Mean [SEM]. The asterisk indicates a p value < 0.05 compared to control group. (**C**) Correlation between tubular damage score and capillary density assessed by RECA1 density in all rats.

As expected MH group experienced a decreased RBF and increased RVR with an aorta remodelling (assessed by an increased intima/media ratio), an increased lumen of interlobar arteries but a decreased lumen of periglomerular arteries altogether with a rarefaction of peritubular capillaries assessed by RECA-1 staining (Figs 4 and 5a,b). Of notice, RBF was also decreased and RVR increased in hemin treated animals (i.e. H and AH rats) with no modification of vessel lumens except for peritubular capillaries which density appears dramatically reduced (especially in AH group). In group A, RBF value was not decreased compared to control group, despite an RVR increase, a decrease of periglomerular arteries lumen and a modest but significant peritubular capillaries rarefaction.

**Figure 6:**
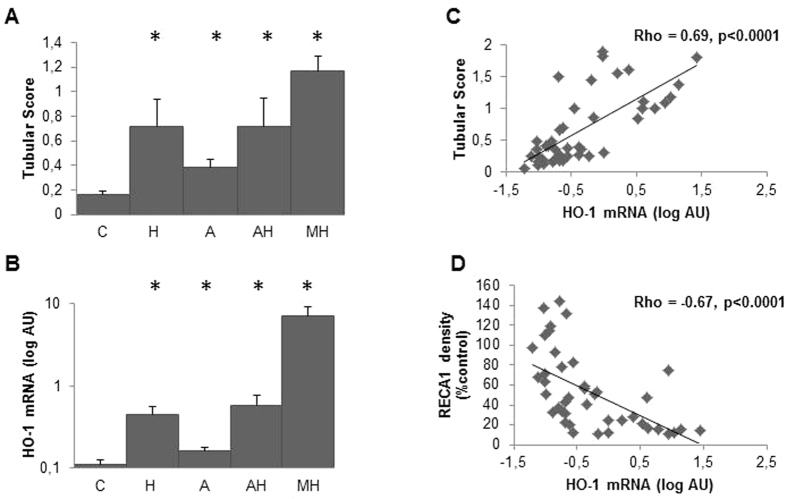
Tubular damage and renal HO-1 expression at day 28. (**A**) Comparison of tubular score between the different groups (C: controls, H: hemin rats, A: angiotensin II rats, AH: angiotensin II and hemin rats, MH: malignant hypertension). Values expressed in Mean [SEM]. (**B**) Comparison of renal HO-1 mRNA expression between the different groups. The asterisk indicates a p value < 0.05 compared to control rats. Correlation between renal HO-1 mRNA expression (**C**) tubular score and (**D**) RECA1 staining (density is expressed as % of controls).

**Figure 7:**
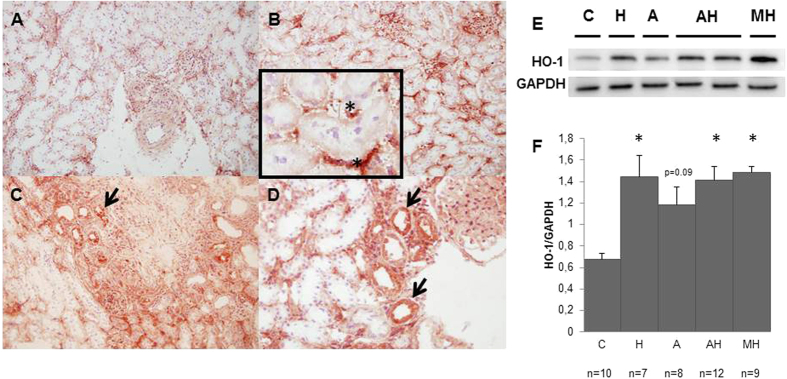
HO-1 expression in kidneys at day 28. Left panel, Representative renal HO-1 staining in a control rat (**A**), hemin rat (**B**), and a rat with malignant hypertension (**C**,**D**). A,B and C: magnification X 200, (**D**): magnification X 400. HO-1 protein expression is upregulated in hemin-treated rats capillaries, and in tubules and capillaries of rats with malignant hypertension. *Indicates HO-1 staining within peritubular capillary, arrow indicates HO-1 tubular staining. Right panel, representative western blot of HO-1 protein (**E**) with mean quantification +/− SEM (HO-1/GAPDH ratio) on whole kidney extracts of all animals included in the study (**F**). The asterisk indicates a p value < 0.05 compared to control rats.

As shown Fig. 5c, peritubular capillaries rarefaction assessed by a renal RECA1 expression decrease was inversely correlated to tubular damage score. Tubular scoring and renal HO1 mRNA levels were higher in MH group but they were also surprisingly high in all treated groups including H group (Fig. 6a and b). Accordingly, renal HO1 mRNA expression was strongly associated with tubular scoring (rho = 0.69, p < 0.0001) (Fig. 6c) and RECA1 staining level (rho = −0.67, p < 0.0001) (Fig. 6e). As shown Fig. 7, HO-1 staining was indeed upregulated within peritubular capillaries in H, AH (not shown) and MH groups whereas RECA1 staining was decreased respectively (Fig. 5). Of notice, a high HO-1 staining within numerous tubules was only detected in MH group (Fig. 7c and d). Western blot analysis performed on whole kidney extracts (Fig. 7e and f) demonstrated a significant increase of HO1 protein expression in MH group (p < 0.001) but also in H and AH groups (p < 0.02 and p = 0.002 respectively).

## Discussion

Our results show that hemin administration worsens angiotensin II mediated vascular nephropathy as assessed by renal hemodynamic and histologic studies. Of notice, renal microvascular impairment and tubular damage were detected even in the absence of angiotensin II and hypertension and plasma heme concentrations in treated rats were in the same order of magnitude as values measured in some patients with chronic hemolysis such as sickle cell disease (personal data). Indeed, whereas high doses of hemin were responsible of heme casts within tubules and animal death due to acute renal failure (data not shown), the issue whether a mild plasma heme increase in the setting of severe hypertension could trigger MH was unknown. Conversely to our working hypothesis, onset of MH was independent of hemin administration among angiotensin II treated animals with an overall occurrence in 45% of cases. Indeed, urine protein/creatinine ratio was significantly higher at day 14 in MH group compared to other groups (Fig. 3), i.e. at an early time point, before randomization (when hemin was not already initiated), thus suggesting that a genetic background heterogeneity, salt intake and angiotensin II doses (uncontrolled differences in AII administration flow through osmotic pumps) are more likely relevant factors triggering MH. On the other hand, a potential preventive effect of hemin also seems unlikely given the detected detrimental effect of hemin administration on vascular and tubular lesions. Moreover, no high protein/creatinine ratio detected at day 14 decreased at day 28 in any AII + hemin animals thus reasonably ruling out a potential beneficial effect of increased plasma heme on glomerular lesions (at least within this concentration range). MH phenotype assessed by histological features at day 28 exhibited a dramatic renal blood flow decrease altogether with expected systemic features such as elevated plasma bilirubin and decreased hematocrit values. Vascular remodeling features were associating interlobar dilation, presumably suggestive of an increased pulse pressure responsible for downstream endothelium damage, including noteworthy glomerular and peritubular capillaries rarefaction assessed by endothelial RECA staining.

Analysis of angiotensin II treated animals with no MH, showed the presence of a high blood pressure with a preserved RBF as expected, despite a RVR increase suggesting effective autoregulatory processes at play. Indeed, aorta remodeling was detected in all the angiotensin II treated rats, in accordance with literature^21^,^22^. Moreover, an expected angiotensin II induced small renal arteries vasoconstriction would explain a controlled downstream blood pressure allowing vessels protection (i.e. glomerular and peritubular capillaries) with a preserved RBF^23^. However, some renal histological lesions and peritubular capillaries rarefaction were detected compared to controls suggesting that a mild endothelial dysfunction was nevertheless occurring.

Conversely, in the AH group with no MH, renal autoregulatory mechanisms appear impaired as RBF and peritubular capillaries were markedly decreased, with significant tubular damage. Noteworthy, AH rats did not display blood pressure or proteinuria changes compared to AII rats at variance with previous studies showing a vasodilator effect of heme (presumably related to carbon monoxide induction)^24^,^25^. Blood pressure values were also similar between rats treated by hemin alone (group H) and controls.

Surprisingly, rats receiving heme alone also displayed a decreased RBF and increased RVR suggesting marked renal damages. Morphometric analyses of renal vessels were thus performed in order to explain these features in hemin rats and ruled out large, medium or small artery morphometric changes, but pointed out a significant peritubular capillary rarefaction assessed by RECA-1 staining. Thus, heme administration alone seems to induce a microangiopathy severe enough to decrease RBF and damage significantly tubules. In agreement with this view, a strong negative correlation was detected between endothelial RECA1 staining and tubular damage score. As a matter of fact, direct endothelial toxicity of heme is consistent with previous reports^2^,^4^,^6^,^26^ and direct toxicity of hemin on tubules was ruled out by Perls staining (except in MH group, data not shown). Furthermore, renal HO-1 mRNA upregulation was strongly correlated with the degree of severity of tubular damage in all treated rats (including hemin groups) though HO-1 protein expression was mostly expressed in peritubular capillaries further strengthening the view that capillaries are the main targets and that vessels rarefaction accounts indeed for RBF decrease. Thus, surprisingly, renal HO-1 quantification appears as a relevant and accurate marker of renal injuries (i.e. vascular and tubular) whereas HO-1 renal expression is supposed to counterbalance oxidative stress to ensure cellular protection and/or adapt heme overload^27^. Accordingly, the presence of a strong HO-1 tubular staining detected only in HM rats appears as a hallmark of a genuine tubular necrosis.

To conclude, plasma heme increase does not trigger angiotensin II malignant hypertension or blood pressure control but damages renal peritubular capillaries leading to increased tubular lesions even in animals not receiving angiotensin II. Thus, our data favor the view that a sustained moderate increased plasma heme similar to the values encountered in chronic hemolysis such as sickle cell disease, but also possibly in some chronic kidney disease patients, induces (or worsens) a microvascular nephropathy. In this setting, occurrence of vascular lesions on larger vessels probably depends both on the magnitude of a sustained renin angiotensin system stimulation and on the duration of free plasma heme exposure.

## Materiel and Methods

### Animals

All procedures were performed in accordance with the French animal care legislation (January 2001), and were approved by the INSERM and Sorbonne University ethic committee. Wild-type male Sprague-Dawley rats (mean weight of 250 g, Harlan Laboratories, Indianapolis, IN, USA) were fed a normal rat diet with free access to water.

### Experimental protocol

Rats were initially randomly allocated into 2 groups: (1) control rats (n = 17), (2) Angiotensin II (Ang II) (n = 29) treated rats. Ang II (400 ng/kg/min) or vehicle was continuously infused into rats aged 8 weeks subcutaneously via an osmotic minipump (Alzet model, Durect Corporation, Cupertino, CA, USA) for 28 days. In the group receiving angiotensin II, NaCl was added to drinking water (at a 6 g/l concentration). Animals were randomized at day 14 to receive either hemin or control. Rats were anesthetized with isoflurane for intraperitoneal injections of hemin (50 mg/kg, Sigma-Aldrich, Saint-Louis, MO, USA) or control (Phosphate Buffer Saline) from day 14 to day 28, thrice a week.

### Hemodynamic Measurements

Systolic BP (SBP) was measured indirectly by the tail-cuff method (CODA System, Kent Scientific, Torrington, CT, USA) twice a week during the 28 days of protocol. At day 28, rats were anesthetized with intraperitoneal pentobarbital (100 μl/100 g), and placed on thermostatically controlled heated surgical table to maintain rectal temperature at 37 °C. The femoral artery was cannulated to allow continuous monitoring of systemic arterial blood pressure and heart rate. The femoral vein was cannulated to allow infusion of solutions. The left kidney was exposed from a laparotomy incision. The renal artery was separated carefully from the renal vein which enabled placement of a flow probe connected with a Transonic flowmeter (Transonic System Inc., Ithaca, NY, USA) for measurement of the total renal blood flow (RBF) and renal vascular resistances (RVR).

### Sample processing

Plasma samples from the above experiments were taken at the moment of sacrifice and were assayed for hematocrit, urea, serum creatinine, lactate dehydrogenase (LDH) and plasma bilirubin by a KONELAB automate (Thermo Scientific, Waltham, MA). Individual rats were housed in metabolic cages and urine was collected over a 4-hour period. This process was repeated four times for each individual (once a week). Urinary protein concentration was normalized to urinary creatinine concentration, and values were expressed as g/mmol creatinuria.

### Morphologic Analysis

Kidneys and aorta were rapidly excised after sacrifice. Aorta sections were rapidly freezed after excision and conserved at −80 °C. Sections 5 μm thick that included the entire circumference were cut from five different blocks of each animal. Kidneys were partly fixed in AFA solution (alcohol-formalin-acetic acid), dehydrated, embedded in paraffin, and further processed for Masson trichrome staining (5 μm sections), and partly freezed with RNA later solution (Qiagen) for subsequent analyses.

#### Morphometric analyses

Digital measurements were blindly performed using the Image J software (NIH, Bethesda, MD, USA). Aortic remodeling was assessed by the averaged measure of intima/media ratio on five sections for each rat. Interlobar arteries and periglomerular arteries of ten representative fields (magnification X 400) were analyzed for each rat (longitudinal renal cutting)^28^. Wall thickness and vascular lumen were measured for each structure. Necrosis was measured in glomeruli and in small vessels of ten representative fields for each rat (magnification X 400)^29^.

#### Tubular Score

Tubular injury was scored by estimating the percentage of tubules that showed tubular dilatation, epithelial necrosis, luminal casts, loss of brush border or naked basement membrane as follows: 0, none; 1, <50%; 2, >50%. The mean tubular damage score was established blindly for each rat on ten representative fields (magnification X 200) of two renal cortical sections.

### Immunofluorescent assay and immunohistochemistry

Snap-frozen kidney samples were processed for direct immunofluorescence microscopy using FITC-labeled antibody specific for fibrin or incubated with anti-RECA1 (ab22492 Abcam, Cambridge, UK) and heme-oxygenase type 1 (HO-1) (ab68477, Abcam, Cambridge, UK) then with appropriate secondary peroxidase labeled antibodies and revealed with AEC (3-amino-9-éthylcarbazole, DakoCytomation, Santa Clara, CA, USA).

### RT-PCR

The renal cortical samples were extracted for total RNA, which were assayed for (HO-1) mRNA by reverse transcription PCR (RT-PCR), the results being normalized to HPRT mRNA.

### Western Blot

Proteins were extracted from renal cortex or isolated glomeruli using RIPA lysis buffer supplemented with sodium orthovanadate, PMSF, a protease inhibitor cocktail (Tebu Bio, Le Perray en Yvelines, France), and 10 mM sodium fluoride. After a centrifugation at 10,000 rpm for 10 min at 4 °C, protein concentrations were determined from the supernatant using the Bradford assay. Aliquots of 10 μg of protein were run on NuPAGE 4/12% electrophoresis gels (Invitrogen, Carlsbad, CA, USA), then transferred onto a PVDF membrane (Millipore, Billerica, MA, USA). Immunoblotting was performed using rabbit specific primary antibodies anti-HO1 (Abcam, Cambridge, UK), and rabbit anti-GAPDH (Sigma Aldrich, Saint Louis, MO, USA) for loading control. Then, the membrane was incubated with horseradish peroxidase-linked donkey secondary antibody (GE Healthcare, Little Chalfont, UK). The revelation was performed with the ECL plus kit (GE Healthcare). Densitometric analysis on ImageJ (NIH, Bethesda, MD, USA) was then performed for quantification.

### Statistical Analysis

All data are presented as percentages or mean ± SEM values. Categorical variables were compared by the Fisher’s exact test when appropriate. As some parameters were not normally distributed, non-parametric Mann-Whitney or Kruskal-Wallis tests as appropriate were used to test differences between groups. Non parametric Spearman tests were used to test associations between variables of interest. The significance level of a statistical hypothesis test was set at 0.05. All statistical analyses were performed using StatView software 5.0 (SAS Institute, Cary, NC, USA).

## Additional Information

**How to cite this article**: Tabibzadeh, N. *et al*. Plasma heme-induced renal toxicity is related to a capillary rarefaction. *Sci. Rep.* **7**, 40156; doi: 10.1038/srep40156 (2017).

**Publisher's note:** Springer Nature remains neutral with regard to jurisdictional claims in published maps and institutional affiliations.

## Figures and Tables

**Table 1:** Biological data at day 28.

Groups	C	H	A	AH	MH
Ht, (%)	54.9 [53.9–57.1]	55.7 [54.6–56.3]	56.8 [55.1–57.2]	58.7 [56.4–63.4]	48.6 [47.8–50.9]*
Plasma heme, (μmol/l)	18.4 [15.6–21.4]	30.4 [26.5–44.6]*	25.5 [24.8–36.2]*	45.1 [30.6–66.9]*	35.9 [27.6–48.9]*
Bilirubin (μmol/l)	1.7 [1.7–1.8]	1.8 [1.8–1.8]	1.8 [1.7–1.9]	2.3 [2.1–2.5]*	2 [1.9–2.8]*
LDH (IU/l)	779 [620–1953]	3844 [3711–4039]	1590 [934–2695]	968 [632–1353]	2042 [1344–2940]

Hematocrit, plasma heme, bilirubin, and LDH levels. Values expressed in Mean [SEM]. C: controls, H: hemin rats, A: angiotensin II rats, AH: angiotensin II and hemin rats, MH: malignant hypertension. Values with an asterisk indicate a p value < 0.05 compared to control rats. No significant difference was found between MH rats and groups A or AH.

## References

[b1] Anderson K. E. . Recommendations for the diagnosis and treatment of the acute porphyrias. Ann. Intern. Med. 142, 439–450 (2005). 15767622 10.7326/0003-4819-142-6-200503150-00010

[b2] Higdon A. N. . Hemin causes mitochondrial dysfunction in endothelial cells through promoting lipid peroxidation: the protective role of autophagy. Am. J. Physiol. Heart Circ. Physiol. 302, H1394–1409 (2012). 22245770 10.1152/ajpheart.00584.2011 PMC3330785

[b3] Jeney V. . Pro-oxidant and cytotoxic effects of circulating heme. Blood 100, 879–887 (2002). 12130498 10.1182/blood.v100.3.879

[b4] Gonzalez-Michaca L., Farrugia G., Croatt A. J., Alam J. & Nath K. A. Heme: a determinant of life and death in renal tubular epithelial cells. Am. J. Physiol. Renal Physiol. 286, F370–377 (2004). 14707007 10.1152/ajprenal.00300.2003

[b5] Balla J. . Endothelial-cell heme uptake from heme proteins: induction of sensitization and desensitization to oxidant damage. Proc. Natl. Acad. Sci. USA 90, 9285–9289 (1993). 8415693 10.1073/pnas.90.20.9285 PMC47552

[b6] Balla J. . Haem, haem oxygenase and ferritin in vascular endothelial cell injury. Nephrol. Dial. Transplant. Off. Publ. Eur. Dial. Transpl. Assoc. - Eur. Ren. Assoc. 18 Suppl 5, v8–12 (2003). 10.1093/ndt/gfg1034 12817058

[b7] Belcher J. D. . Heme triggers TLR4 signaling leading to endothelial cell activation and vaso-occlusion in murine sickle cell disease. Blood 123, 377–390 (2014). 24277079 10.1182/blood-2013-04-495887 PMC3894494

[b8] Camus S. M. . Circulating cell membrane microparticles transfer heme to endothelial cells and trigger vasoocclusions in sickle cell disease. Blood 125, 3805–3814 (2015). 25827830 10.1182/blood-2014-07-589283 PMC4490297

[b9] Latunde-Dada G. O., Simpson R. J. & McKie A. T. Recent advances in mammalian haem transport. Trends Biochem. Sci. 31, 182–188 (2006). 16487711 10.1016/j.tibs.2006.01.005

[b10] Nath K. A. & Hebbel R. P. Sickle cell disease: renal manifestations and mechanisms. Nat. Rev. Nephrol. 11, 161–171 (2015). 25668001 10.1038/nrneph.2015.8 PMC4701210

[b11] Nath K. A. & Katusic Z. S. Vasculature and kidney complications in sickle cell disease. J. Am. Soc. Nephrol. JASN 23, 781–784 (2012). 22440903 10.1681/ASN.2011101019 PMC3338300

[b12] Csongradi E., Juncos L. A., Drummond H. A., Vera T. & Stec D. E. Role of carbon monoxide in kidney function: is a little carbon monoxide good for the kidney? Curr. Pharm. Biotechnol. 13, 819–826 (2012). 22201605 10.2174/138920112800399284 PMC3354025

[b13] Frei P. . Liver Transplantation because of Acute Liver Failure due to Heme Arginate Overdose in a Patient with Acute Intermittent Porphyria. Case Rep. Gastroenterol. 6, 190–196 (2012). 22649331 10.1159/000338354 PMC3362186

[b14] Dhar G. J., Bossenmaier I., Cardinal R., Petryka Z. J. & Watson C. J. Transitory renal failure following rapid administration of a relatively large amount of hematin in a patient with acute intermittent porphyria in clinical remission. Acta Med. Scand. 203, 437–443 (1978). 665312 10.1111/j.0954-6820.1978.tb14903.x

[b15] Ryter S. W., Otterbein L. E., Morse D. & Choi A. M. K. Heme oxygenase/carbon monoxide signaling pathways: regulation and functional significance. Mol. Cell. Biochem. 234–235, 249–263 (2002). 10.1023/A:1015957026924 PMC7101540 12162441

[b16] Zager R. A., Johnson A. C. M. & Becker K. Plasma and urinary heme oxygenase-1 in AKI. J. Am. Soc. Nephrol. JASN 23, 1048–1057 (2012). 22440905 10.1681/ASN.2011121147 PMC3358765

[b17] Botros F. T., Dobrowolski L. & Navar L. G. Renal heme oxygenase-1 induction with hemin augments renal hemodynamics, renal autoregulation, and excretory function. Int. J. Hypertens. 2012, 189512 (2012). 22518281 10.1155/2012/189512 PMC3296275

[b18] Wang R., Shamloul R., Wang X., Meng Q. & Wu L. Sustained normalization of high blood pressure in spontaneously hypertensive rats by implanted hemin pump. Hypertension 48, 685–692 (2006). 16940218 10.1161/01.HYP.0000239673.80332.2f

[b19] Tracz M. J. . Deficiency of heme oxygenase-1 impairs renal hemodynamics and exaggerates systemic inflammatory responses to renal ischemia. Kidney Int. 72, 1073–1080 (2007). 17728706 10.1038/sj.ki.5002471 PMC2948968

[b20] Yachie A. . Oxidative stress causes enhanced endothelial cell injury in human heme oxygenase-1 deficiency. J. Clin. Invest. 103, 129–135 (1999). 9884342 10.1172/JCI4165 PMC407858

[b21] Griffin S. A. . Angiotensin II causes vascular hypertrophy in part by a non-pressor mechanism. Hypertension 17, 626–635 (1991). 2022407 10.1161/01.hyp.17.5.626

[b22] Dzau V. J., Gibbons G. H. & Pratt R. E. Molecular mechanisms of vascular renin-angiotensin system in myointimal hyperplasia. Hypertension 18, II100–105 (1991). 1916996 10.1161/01.hyp.18.4_suppl.ii100

[b23] Arendshorst W. J., Brännström K. & Ruan X. Actions of angiotensin II on the renal microvasculature. J. Am. Soc. Nephrol. JASN 10 Suppl 11, S149–161 (1999). 9892156

[b24] Jadhav A., Torlakovic E. & Ndisang J. F. Hemin therapy attenuates kidney injury in deoxycorticosterone acetate-salt hypertensive rats. Am. J. Physiol. Renal Physiol. 296, F521–534 (2009). 19116243 10.1152/ajprenal.00510.2007

[b25] Shamloul R. & Wang R. Monitoring circulatory heme level in hemin therapy for lowering blood pressure in rats. Cell. Mol. Biol. Noisy–Gd. Fr. 51, 507–512 (2005). 16309573

[b26] Vinchi F. . Hemopexin therapy improves cardiovascular function by preventing heme-induced endothelial toxicity in mouse models of hemolytic diseases. Circulation 127, 1317–1329 (2013). 23446829 10.1161/CIRCULATIONAHA.112.130179

[b27] Nath K. A. . Heme protein-induced chronic renal inflammation: suppressive effect of induced heme oxygenase-1. Kidney Int. 59, 106–117 (2001). 11135063 10.1046/j.1523-1755.2001.00471.x

[b28] Vinsonneau C. . Intrarenal urothelium proliferation: an unexpected early event following ischemic injury. Am. J. Physiol. Renal Physiol. 299, F479–486 (2010). 20591940 10.1152/ajprenal.00585.2009

[b29] Kerroch M. . Genetic inhibition of discoidin domain receptor 1 protects mice against crescentic glomerulonephritis. FASEB J. Off. Publ. Fed. Am. Soc. Exp. Biol. 26, 4079–4091 (2012). 10.1096/fj.11-194902 22751008

